# Assessment of the Effectiveness of Identity-Based Public Health Announcements in Increasing the Likelihood of Complying With COVID-19 Guidelines: Randomized Controlled Cross-sectional Web-Based Study

**DOI:** 10.2196/25762

**Published:** 2021-04-13

**Authors:** Alexander S Dennis, Patricia L Moravec, Antino Kim, Alan R Dennis

**Affiliations:** 1 Smith School of Business University of Maryland College Park, MD United States; 2 McCombs School of Business University of Texas at Austin Austin, TX United States; 3 Kelley School of Business Indiana University Bloomington, IN United States

**Keywords:** Amazon Mechanical Turk, compliance, COVID-19, custom, effectiveness, guideline, identity, public health, public health announcement, public service announcement, social media, web-based health information

## Abstract

**Background:**

Public health campaigns aimed at curbing the spread of COVID-19 are important in reducing disease transmission, but traditional information-based campaigns have received unexpectedly extreme backlash.

**Objective:**

This study aimed to investigate whether customizing of public service announcements (PSAs) providing health guidelines to match individuals’ identities increases their compliance.

**Methods:**

We conducted a within- and between-subjects, randomized controlled cross-sectional, web-based study in July 2020. Participants viewed two PSAs: one advocating wearing a mask in public settings and one advocating staying at home. The control PSA only provided information, and the treatment PSAs were designed to appeal to the identities held by individuals; that is, either a Christian identity or an economically motivated identity. Participants were asked about their identity and then provided a control PSA and treatment PSA matching their identity, in random order. The PSAs were of approximately 100 words.

**Results:**

We recruited 300 social media users from Amazon Mechanical Turk in accordance with usual protocols to ensure data quality. In total, 8 failed the data quality checks, and the remaining 292 were included in the analysis. In the identity-based PSA, the source of the PSA was changed, and a phrase of approximately 12 words relevant to the individual’s identity was inserted. A PSA tailored for Christians, when matched with a Christian identity, increased the likelihood of compliance by 12 percentage points. A PSA that focused on economic values, when shown to individuals who identified as economically motivated, increased the likelihood of compliance by 6 points.

**Conclusions:**

Using social media to deliver COVID-19 public health announcements customized to individuals’ identities is a promising measure to increase compliance with public health guidelines.

**Trial Registration:**

ISRCTN Registry 22331899; https://www.isrctn.com/ISRCTN22331899.

## Introduction

Public compliance with recommended guidelines to limit the spread of SARS-CoV-2 and COVID-19 is an important component in combating the disease [1]. Current guidelines suggest several measures, such as wearing a mask and staying at home [2]; nonetheless, a large number of individuals fail to follow the guidelines provided by public health officials [3]. Public compliance to guidelines remains an issue [3-5].

Public service announcements (PSAs) have long been used to promote public health behaviors, although their success and the success of PSAs in general have been inconsistent [6]. Information-based PSAs are often successful because they present facts about the nature of a threat, explain the benefits of a response, and provide a clear call to action [7]. However, it remains unclear whether presenting information is sufficient in a posttruth era as the world battles the COVID-19 pandemic. There has been strong backlash against wearing masks and staying at home [3,5] and “irrational behavior” in noncompliance with COVID-19 policies [8]. Psychological reactance occurs when individuals feel that previously permitted behaviors are constrained by an external agent, which impugns their freedom [9,10]. In such situations, individuals resist the constraint and attempt to regain their lost freedom [9,10]. Thus, rather than helping, a PSA could backfire by sparking reactance, which triggers individuals to eschew the recommended behavior and even actively impair compliance [11].

The likelihood of reactance may also be increased by the divisions among people in the United States along ideological lines, with stark differences in the extent to which those on the political left and right wings believe that COVID-19 is a legitimate threat [1,12-14]. In the era of tribalism and distrust toward experts, identity has become as central to many arguments as scientific information [14-16]. The interaction of an individual’s identity with the source and content of the message can shape responses as much as the information that the message contains [17,18].

Individuals’ identities determine how they answer the question of “who am I?” [19-21]. According to the social identity theory, these answers are dependent upon both social and personal identities [19-22]. Social identities derive from the social groups to which individuals belong [22], such as race, nationality, and organizational or religious affiliations [23-25]. Personal identities derive from values that individuals consider important [20,25,26], such as volunteering [27], environmentalism [28], or the economy and economic values [29-31]. Both social and personal identities can be potent influences on behavior because people are motivated to act in ways that align with their identity in order to maintain a sense of self-consistency [22,25,32,33].

Persuasive messages such as PSAs can take advantage of this desire for identity-consistent actions by framing a proposed action as being consistent with individuals’ social or personal identity [34]. Speaking the language of an identity by using terms and arguments associated with that identity may render a more persuasive message [35]. Framing the advocated action as identity-consistent can further encourage individuals to adopt the desired behavior [34] because once an individual knows how others with the same identity act, it is easier to convince oneself to act in that same manner [1]. Thus, identity-framed messages are more persuasive than general messages [35].

This study aimed to investigate whether customizing PSAs in accordance with the source and language of a social identity (specifically Christian) or a personal identity (specifically economically motivated) increases the intention of individuals who identify with those identities to comply with the behaviors advocated by a PSA. We selected these identities because individuals who resist public health guidelines frequently provide religious [36,37] and economic [38] excuses. If individuals with these identities could be persuaded to follow public health guidelines, the benefits could be substantial [39]. We sought to investigate whether framing a PSA in accordance with a Christian social identity or an economically motivated personal identity increases the likelihood of compliance with COVID-19 guidelines among individuals who hold those identities.

## Methods

### Study Overview

We conducted a within- and between-subjects, randomized controlled cross-sectional, web-based study. All data were collected on the internet and no identifying information was collected in order to protect participants’ privacy and confidentiality. The study was reviewed by the institutional review board of Indiana University (protocol# 2004499544) and was determined to be an exempt study. The study was initiated with the institutional review board approving the study data and if participants consented to participate, they were enrolled in the study.

### Participants

In July, 2020, we recruited 300 participants from Amazon Mechanical Turk in accordance with the usual protocols to ensure data quality [40]. We recruited participants only from the United States who held an Amazon Masters classification and included a captcha to preclude nonhuman responses. Participants were paid US $1.25 and spent an average of about 13 minutes participating in the study (minimum: 3.6 minutes, maximum 11 hours). In total, 8 subjects failed 1 or more of the 3 attention checks (that asked participants to select specific answers), thus yielding 292 participants. All participants received both the control and treatment conditions; hence, demographics are described at the study level. Approximately 49% of participants were female, and 82% were White, 8% were Asian, 7% were Black, and 3% were of other racial backgrounds. The median age of the study participants was 30 years (24%: 18-24 years, 33%: 25-34 years, 19%: 35-44 years, 16%: 45-54 years, 6%: 55-64 years, and 1%: ≥65 years).

### Study Design and Interventions

**Figure 1:**
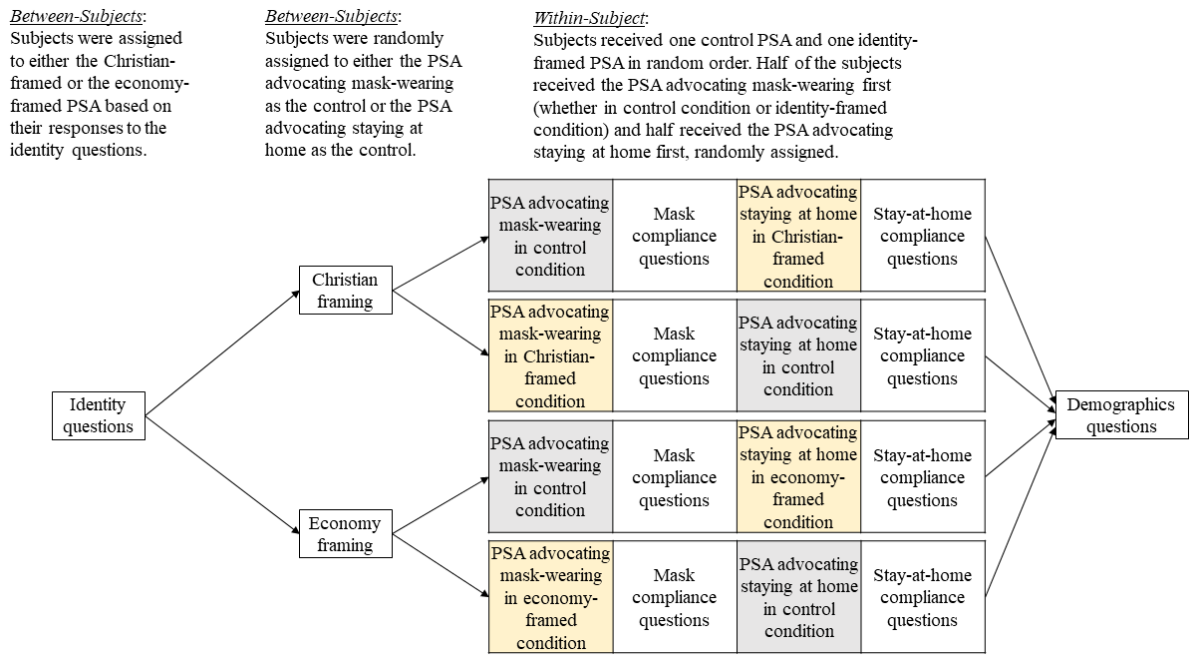
Experimental design. PSA: public service announcement.

At the beginning of the survey, subjects were asked a series of questions to ascertain their identification with Christianity and with the economic health of the country (our selected identities). Participants were provided either the Christian-framed treatment or economy-framed PSAs depending on which identity they identified with more. If subjects identified with both identities equally, they were randomly assigned to 1 of the 2 treatments. The experimental design is presented in Figure 1.

Participants then received two COVID-19 PSAs in random order: 1 advocating wearing a mask and 1 calling on people to stay at home. One was a control PSA with information purportedly from the US Public Health Service and the other was an identity-framed PSA (either Christian-framed or economy-framed). After reading each PSA, participants reported the extent to which they would engage in the advocated behavior. Each subject received 1 identity-framed PSA and 1 control PSA; thus, we could examine the within-person effects of PSA framing and could control for differences in compliance between mask-wearing and staying at home.

The PSAs were of approximately 100 words. The treatments changed the source of the PSA and 1 sentence of their content. The identity-framed PSA contained a single short, substituted phrase designed to appeal either to people who held a Christian social identity (purportedly written by the Chaplain of the US Senate) or to those for whom protecting the country’s economy was a central feature of their personal identity (purportedly written by the US Chamber of Commerce). For example, the control PSA for wearing a mask stated, “You should wear a mask whenever you are in public and see other people,” which was replaced by, “We have a Christian duty to love our neighbors, and wearing a mask whenever you are in public and see other people is a way you can do this” in the Christian-framed PSA and by, “We now know how you can do your part to help us safely reopen our economy: wearing a mask whenever you are in public and see other people” in the economy-framed PSA. All PSAs are provided in Multimedia Appendix 1.

### Measurements

In accordance with previous studies, Christian identity, economically motivated identity, and trust in the 3 PSA sources were measured with single-item 7-point Likert scales [41-43]. Identity-framed PSAs were deemed to be aligned with the participant’s identity when the participant’s identity scores were 6 or 7 and the participant did not distrust the source (ie, trust in the source was ≥4). The likelihood of compliance was measured using 7 items adapted from previous studies [44,45]. The outcome compliance items were measured using scales of 0-100, not the same 7-point scales as those used for the independent variables to classify participants, to reduce the risk of a common method bias [46]. The likelihood of compliance proved reliable (Cronbach *α*=.94), thus indicating convergent validity. An exploratory factor analysis was performed to assess the discriminant validity among the constructs, which was satisfactory. The results of the items and factor analysis are presented in Multimedia Appendix 1.

### Statistical Analysis

A power analysis using G*Power [47] determined that a sample of 300 participants with this design would provide a power of .93 to detect a small effect size (Cohen *f*=0.10). We analyzed the data using hierarchical linear modeling (HLM) [48] with robust standard errors using HLM for Windows (version 6.00, Scientific Software International, Inc). HLM accounts for the correlation among the repeated within-subject observations and facilitates the assessment of the alignment or nonalignment of the identity-framed treatment condition with the participant’s identity. All tests were 2-tailed with a significance level of α=.05 *α*=.05. We used the formula of Snijders and Bosker [49] to calculate R^2^. The likelihood of complying was nonnormally distributed. For the PSA advocating mask-wearing, the mean likelihood of compliance was 78.75 (SD 29.79), and the median likelihood was 92.93; for the PAS advocating staying at home, the mean likelihood of compliance was 74.51 (SD 29.67), and the median likelihood was 86.75. HLM is robust to departures from the normality assumption for large samples such as this one [48].

## Results

Table 1 shows the mean (SD) values of the likelihood of compliance under different experimental conditions. Overall, participants displayed more willingness to wear a mask than to stay at home. Furthermore, we observed higher means for identity-aligned PSAs than for non–identity-aligned PSAs. Table 2 shows the results of statistical analysis. The overall model has a large effect size, with an R^2^ of 41.6%.

When participants received an identity-framed PSA that was aligned with their identity, they were more likely to comply with it rather than a purely information-based PSA in the control treatment (Christian-framed PSA: *P*=.01; economy-framed PSA: *P*=.01). The effects were significant, increasing compliance by almost 13% to the Christian-framed PSA (95% CI 2.9-22.6) and almost 7% to the economy-framed PSA (95% CI 1.5-12.1) compared to the control non–identity-framed PSA. The average effect sizes (Cohen *d*) for the Christian-framed and economy-framed PSAs were 0.30 and 0.24, respectively, which are between small and medium. This is congruent with our predictions that providing individuals with customized PSAs that align with their identities will increase their intention to comply with the advocated behaviors including staying at home or wearing a mask in public.

When participants received an identity-framed PSA that was not aligned with their identity, it did not significantly influence their likelihood of complying, although both nonaligned PSAs approached significance with negative coefficients (Christian-framed PSA: *P*=.10; economy-framed PSA: *P*=.10), which suggests that a nonaligned PSA may potentially be more damaging to compliance than a control PSA. Compliance was significantly greater for PSAs advocating mask-wearing than for those advocating staying at home (*P*=.001), which suggests that our participants were more likely to comply with the practice of wearing a mask than staying at home. Likewise, the main effects of some of the other control variables that we used to assess alignment (ie, trust and identity) were significant and some were not.

**Table 1:** Means for the likelihood of complying with public service announcements (PSAs). Data were collected in July 2020 from 292 participants from Amazon Mechanical Turk, who viewed two PSAs: 1 advocating wearing a mask and 1 advocating staying at home. One PSA was an information-based control PSA and the other was a Christian or economically motivated identity–framed PSA.

PSA type	Likelihood of staying at home			Likelihood of wearing a mask	
	Mean (SD)	Number of participants	Mean (SD)		Number of participants
Economy-framed PSA when aligned	78.94 (21.54)	65	84.26 (19.68)		78
Christian-framed PSA when aligned	81.99 (26.29)	31	84.62 (27.97)		24
Nonaligned PSA	68.09 (34.59)	51	63.35 (37.76)		43
Control PSA	71.09 (31.11)	145	77.85 (30.85)		147

**Table 2:** Analysis results with beta coefficients (β) for identity-framed public service announcements (PSAs) and information-based control PSAs (584 observations; 292 participants; R^2^=41.6%).

Parameter			Likelihood of complying with PSAs		
			*β* (SE)		*P* value
Economy-framed PSA			−3.60 (2.19)		.10
PSA aligned with an economically motivated identity			6.77 (2.65)		.01
Christian-framed PSA			−6.86 (4.11)		.10
PSA aligned with a Christian identity			12.74 (4.94)		.01
PSA advocating mask-wearing			4.06 (1.23)		.001
Christian identity			−1.60 (0.64)		.01
Economically-motivated identity			−2.56 (1.41)		.07
**Measure of trust**					
	Trust in the US Public Health Service	12.40 (1.12)		.00	
	Trust in the Senate Chaplain	−0.12 (0.64)		.92	
	Trust in the Chamber of Commerce	−1.42 (1.21)		.24	
Constant			74.02 (1.61)		.00

## Discussion

### Principal Findings

Our study shows that modifying PSAs to leverage social and personal identities can promote increased compliance with public health guidelines for individuals who hold these identities. The Christian-framed PSA increased compliance by approximately 12.74 points (out of 100) when viewed by those with a Christian identity, and the economy-framed PSA increased compliance by approximately 6.77 points when viewed by those with an economically motivated identity. One might conclude that the Christian framing is more powerful, but this only applies to individuals who hold that identity. Hence, we believe that it is better to compare the 2 different identity-framed PSAs to their own controls, not to each other, as the differences in the coefficients between the 2 identity-aligned PSAs may have resulted from various subject-level factors that influence their different identities. For example, those with a Christian identity were slightly less likely to comply (*P*=.01) with any PSA, possibly because those with conservative beliefs are less likely to believe that COVID-19 is a legitimate threat [1,14].

Based on the social identity theory, we hypothesized that messages designed to activate an identity by using inclusive language and a consistent message source would be more effective in increasing compliance to PSAs rather than those without consistent identity-framing. Identity-framing was intended to emphasize commonalities between the individual and like-group members and to encourage users to act in accordance with those in their group [1]. Identity-framing harnesses the relevant identity that the individual holds and appeals to those relevant traits. This simple act of creating identity-aligned targeted PSAs significantly increased compliance with the behavior advocated in the PSAs. We decided to examine a Christian identity and an economically motivated identity on the basis of excuses that are commonly invoked to justify noncompliance [36-38]. We found both to be effective in increasing compliance. Various other social or personal identities may also be effective in increasing the compliance to PSAs.

The promise of identity-framed PSAs is noteworthy in view of the myriad of rampant rumors, misinformation, and disinformation regarding COVID-19 [12-14]. Rapidly developing situations, uncertainty, and fear foster the spread of false information (created with or without the deliberate intention to mislead people). It is unfortunate that individuals’ responses to COVID-19 have implied that the provision of simple, information-based PSAs sometimes triggered psychological reactance and led to actions that disrupt public health efforts [1,13]. Our results show that designing PSAs to appeal to specific target demographics can increase their effectiveness beyond that of a message that simply provides correct information.

The vast amount of information about individuals available on social media platforms makes it practical to create multiple versions of a PSA and share the most individually relevant version with people, thereby making the message more persuasive [50]. Social media is an attractive channel to rapidly reach many people as it has more than 2 billion active users [50]. Identity-framed PSAs could facilitate public health goals by enabling the PSA to influence people who would otherwise ignore the message, capturing their attention by speaking their language, activating relevant identities, and reducing psychological reactance by framing the actions as being consistent with their identity [25,35]. By encouraging people to view a situation through the lens of a supportive identity, the effects of countervailing identities that dissuade people from the desired outcome can be reduced. Public Health agencies and nonprofits should take advantage of these tools when designing future public awareness campaigns. By leveraging readily available identity information to make minor adjustments to the framing of PSAs, such groups could facilitate higher compliance with public health guidelines, thus enabling better outcomes. With increasing ease of accessing personal data, the small cost of targeting a PSA toward those individuals that would best respond (similar to targeted advertising) has the potential to yield enormous benefits with increased nationwide health outcomes.

We examined 1 social identity (Christian) and 1 personal identity (economically motivated) linked to noncompliance [36-38]. Many other identities may also be leveraged to enhance the effectiveness of PSAs. By reminding people of a role identity as a parent or grandchild, leveraging social group identities such as sport team loyalty, or by appealing to their self-identity as caring individuals, many opportunities are available to use identities to help persuade people to follow public health guidelines. Our use of 2 identities shows that this method can be successful; however, it is unclear which other identities may also be used. Future studies are required to investigate why some identities may be effective when used to increase compliance while others may not be effective. Furthermore, it remains unclear whether compliance is affected more by the relationship between the identity and the target behavior or that between the identity and the individual, or whether both relationships are equally important.

While we control for trust toward the source of the PSA, a potentially interesting question is one regarding the mediating influence of variables (eg, perceived similarity to the identity used in the PSA). While we did not assess perceived similarity, we controlled for the strength of the identity. Since our study suggests that identity-framed PSAs can be used to influence compliance, more studies are needed to investigate the potential mediating and moderating variables that could strengthen or weaken the effectiveness of identity-framed PSAs. For example, our identity-framed PSA contained only 1 modified sentence; however, it remains unclear whether adding more identity-related framing would increase its effectiveness, or whether it is sufficient to simply invoke the identity as in our PSAs. Moreover, it remains unclear whether the source of the message is a critical factor, or whether the message more important than the source.

### Limitations

This study has the usual limitations of randomized controlled cross-sectional study. We assessed self-reported perceptions at one point in time, not actual behavior over several periods of time. Our participants were those who participate in research studies and thus may differ from those who decline to participate in research studies.

### Conclusions

Compliance with public health measures designed to mitigate the COVID-19 pandemic has unfortunately become intertwined with identity, and individuals with certain social and personal identities are less likely to comply with the behaviors advocated in the PSAs. Our study shows that identity can also be an effective factor to induce compliance. The development of identity-framed PSAs may be effective in contexts beyond the COVID-19 pandemic. The most important factors to consider when developing effective PSAs are that the identities in question are deeply held and can be associated with recommended actions when coordinated efforts across society are needed.

## Appendix

Multimedia Appendix 1 Experimental Materials.

Multimedia Appendix 2 CONSORT-EHEALTH checklist (V 1.6.1).

## Abbreviations

HLM: hierarchical linear modeling
PSA: public service announcement
