# Correction: Assessment of the Effectiveness of Identity-Based Public Health Announcements in Increasing the Likelihood of Complying With COVID-19 Guidelines: Randomized Controlled Cross-sectional Web-Based Study

**DOI:** 10.2196/29603

**Published:** 2021-04-15

**Authors:** Alexander S Dennis, Patricia L Moravec, Antino Kim, Alan R Dennis

**Affiliations:** 1 Smith School of Business University of Maryland College Park, MD United States; 2 McCombs School of Business University of Texas at Austin Austin, TX United States; 3 Kelley School of Business Indiana University Bloomington, IN United States

In “Assessment of the Effectiveness of Identity-Based Public Health Announcements in Increasing the Likelihood of Complying With COVID-19 Guidelines: Randomized Controlled Cross-sectional Web-Based Study” (JMIR Public Health Surveill 2021;7(4):e25762) the authors noted one error.

In the originally published manuscript, an Editorial Notice regarding the study’s retrospective registration contained an incorrect quotation from the authors.

This randomized study was only retrospectively registered, explained by the authors with “this is a replication study”. The editor granted an exception from ICMJE rules mandating prospective registration of randomized trials because the risk of bias appears low and the study was considered formative, guiding the development of the application, or specific requirements regarding preregestration. However, readers are advised to carefully assess the validity of any potential explicit or implicit claims related to primary outcomes or effectiveness, as retrospective registration does not prevent authors from changing their outcome measures retrospectively.

The corrected version of this notice reads as follows:

This randomized study was only retrospectively registered as, according to the authors, their field has not adopted pre-registration as convention. The editor granted an exception from ICMJE rules mandating prospective registration of randomized trials because the risk of bias appears low. However, readers are advised to carefully assess the validity of any potential explicit or implicit claims related to primary outcomes or effectiveness, as retrospective registration does not prevent authors from changing their outcome measures retrospectively.

The correction will appear in the online version of the paper on the JMIR Publications website on April 15, 2021, together with the publication of this correction notice. Because this was made after submission to PubMed, PubMed Central, and other full-text repositories, the corrected article has also been resubmitted to those repositories.

