# Supplementary material for: Assessment of the Effectiveness of Identity-Based Public Health Announcements in Increasing the Likelihood of Complying With COVID-19 Guidelines: Randomized Controlled Cross-sectional Web-Based Study
Source: JMIR Public Health Surveill. 2021 Apr 13;7(4):e25762. doi: 10.2196/25762 (PMC8045778; doi:10.2196/25762)
Supplement: Multimedia Appendix 1 [file publichealth_v7i4e25762_app1.pptx]

## Slide 1
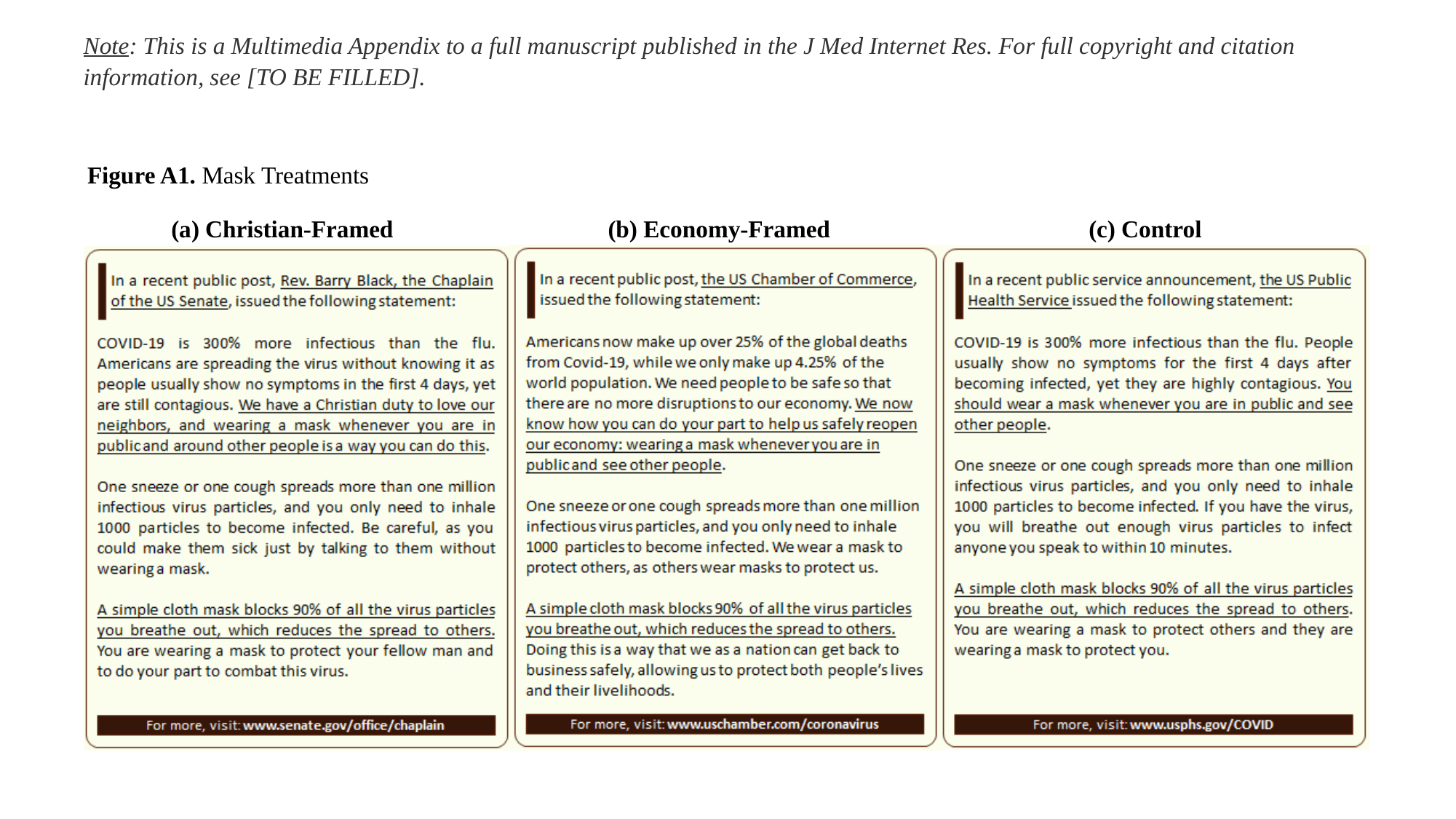

Note: This is a Multimedia Appendix to a full manuscript published in the J Med Internet Res. For full copyright and citation information, see [TO BE FILLED].
Figure A1. Mask Treatments
(a) Christian-Framed
(b) Economy-Framed
(c) Control

## Slide 2
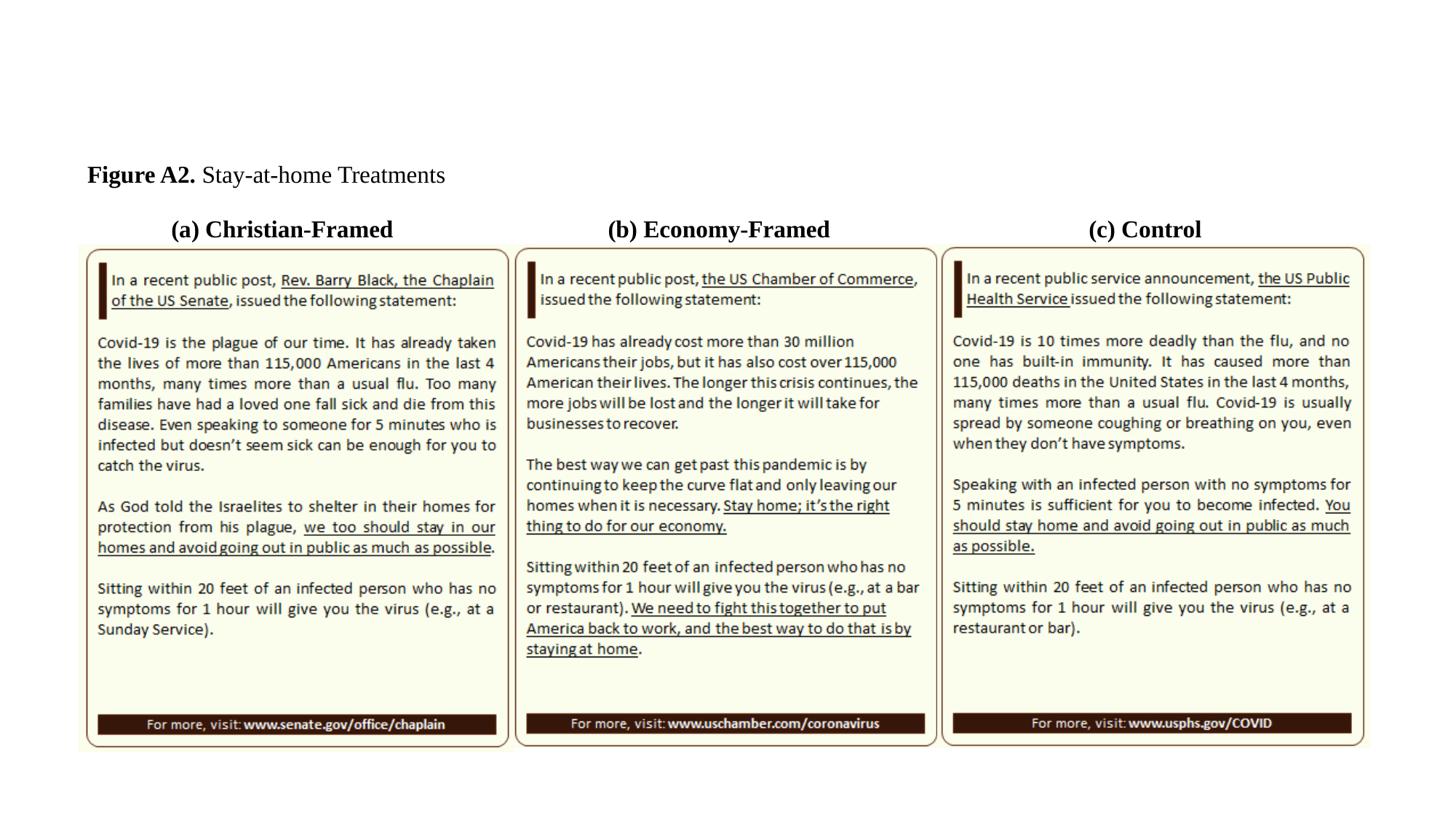

Figure A2. Stay-at-home Treatments
(a) Christian-Framed
(b) Economy-Framed
(c) Control

## Slide 3
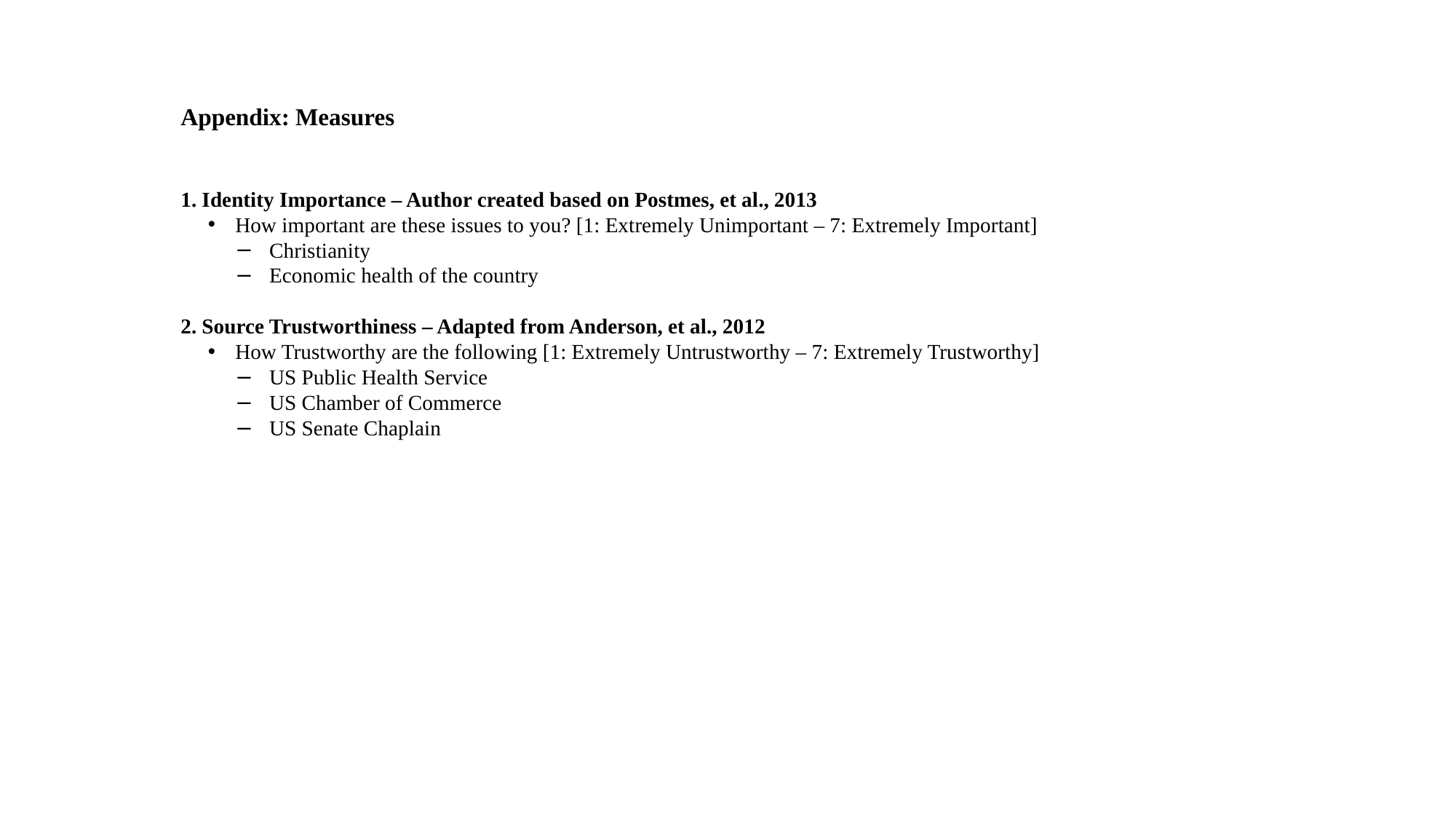

Appendix: Measures
1. Identity Importance – Author created based on Postmes, et al., 2013
How important are these issues to you? [1: Extremely Unimportant – 7: Extremely Important]
Christianity
Economic health of the country
2. Source Trustworthiness – Adapted from Anderson, et al., 2012
How Trustworthy are the following [1: Extremely Untrustworthy – 7: Extremely Trustworthy]
US Public Health Service
US Chamber of Commerce
US Senate Chaplain

## Slide 4
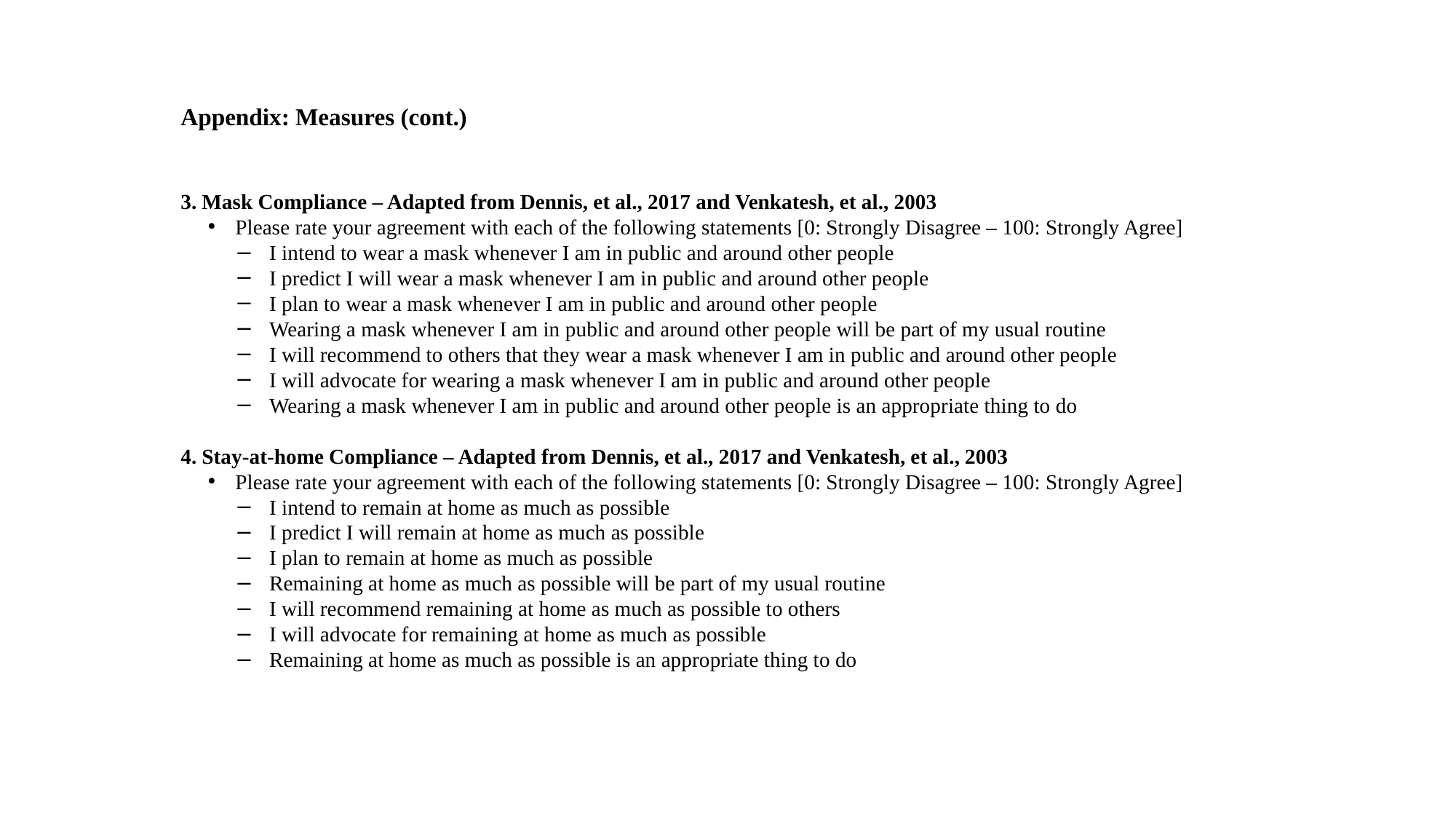

Appendix: Measures (cont.)
3. Mask Compliance – Adapted from Dennis, et al., 2017 and Venkatesh, et al., 2003
Please rate your agreement with each of the following statements [0: Strongly Disagree – 100: Strongly Agree]
I intend to wear a mask whenever I am in public and around other people
I predict I will wear a mask whenever I am in public and around other people
I plan to wear a mask whenever I am in public and around other people
Wearing a mask whenever I am in public and around other people will be part of my usual routine
I will recommend to others that they wear a mask whenever I am in public and around other people
I will advocate for wearing a mask whenever I am in public and around other people
Wearing a mask whenever I am in public and around other people is an appropriate thing to do
4. Stay-at-home Compliance – Adapted from Dennis, et al., 2017 and Venkatesh, et al., 2003
Please rate your agreement with each of the following statements [0: Strongly Disagree – 100: Strongly Agree]
I intend to remain at home as much as possible
I predict I will remain at home as much as possible
I plan to remain at home as much as possible
Remaining at home as much as possible will be part of my usual routine
I will recommend remaining at home as much as possible to others
I will advocate for remaining at home as much as possible
Remaining at home as much as possible is an appropriate thing to do

## Slide 5
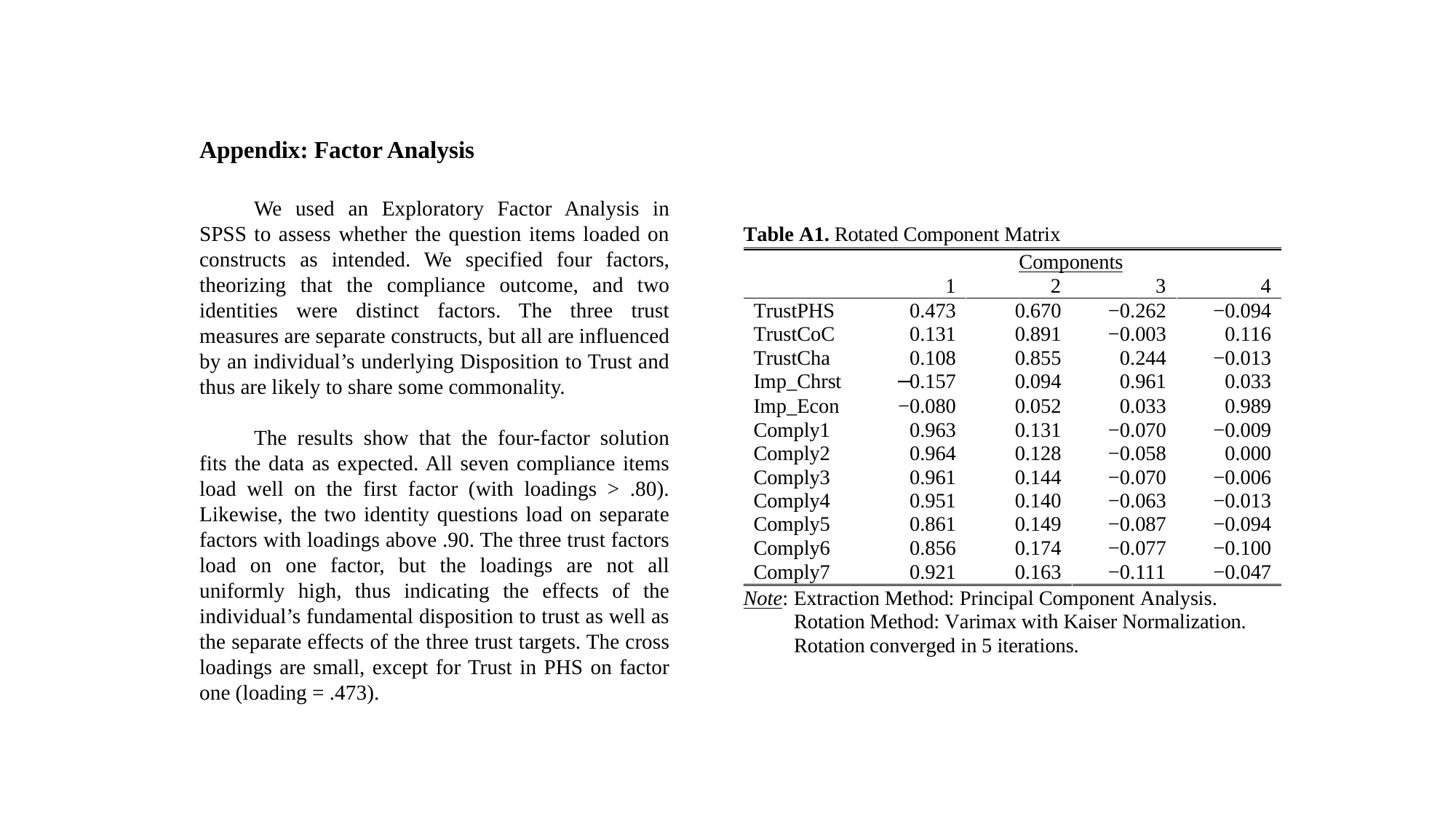

Appendix: Factor Analysis
We used an Exploratory Factor Analysis in SPSS to assess whether the question items loaded on constructs as intended. We specified four factors, theorizing that the compliance outcome, and two identities were distinct factors. The three trust measures are separate constructs, but all are influenced by an individual’s underlying Disposition to Trust and thus are likely to share some commonality.
The results show that the four-factor solution fits the data as expected. All seven compliance items load well on the first factor (with loadings > .80). Likewise, the two identity questions load on separate factors with loadings above .90. The three trust factors load on one factor, but the loadings are not all uniformly high, thus indicating the effects of the individual’s fundamental disposition to trust as well as the separate effects of the three trust targets. The cross loadings are small, except for Trust in PHS on factor one (loading = .473).

## Slide 6
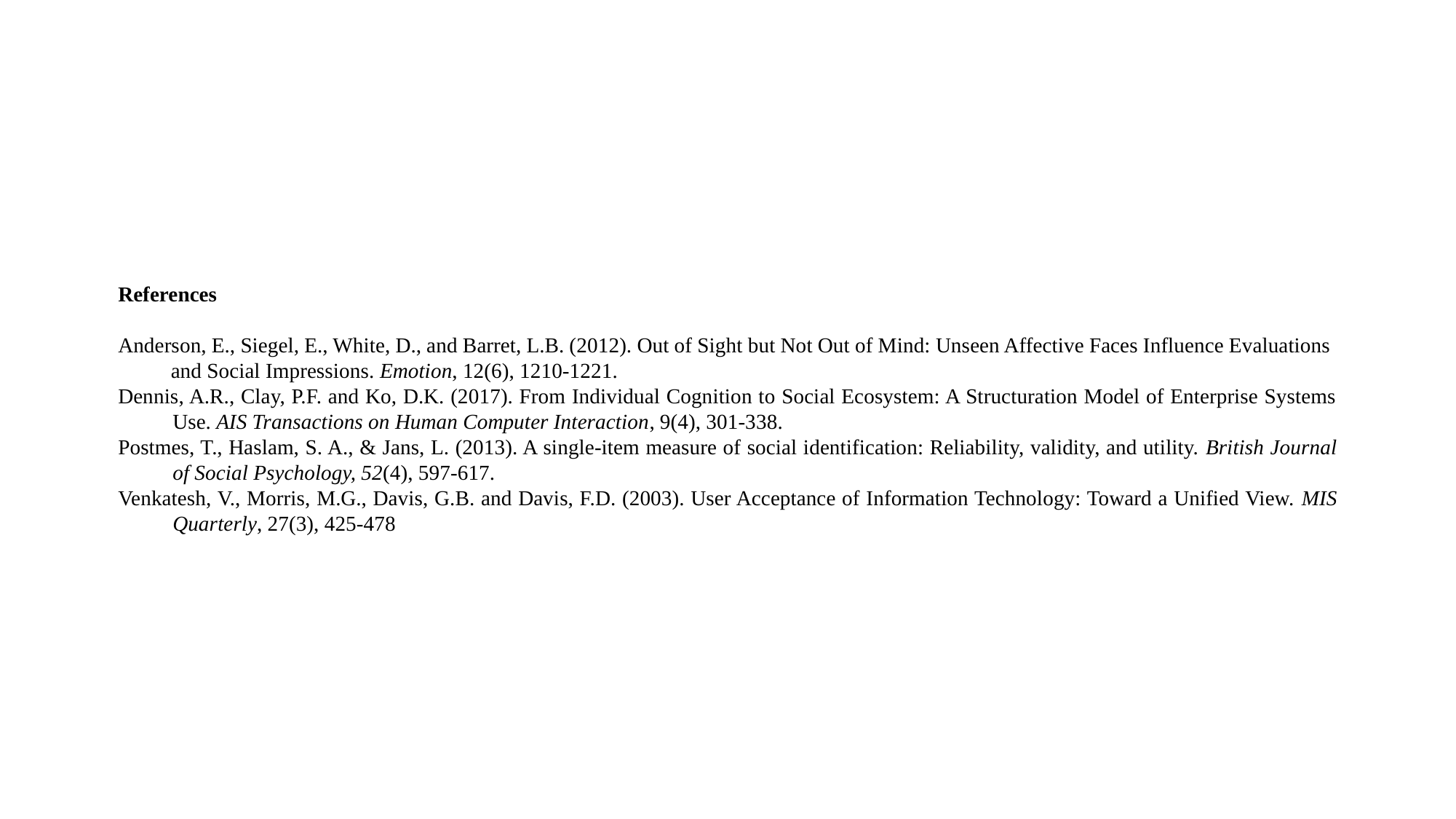

References
Anderson, E., Siegel, E., White, D., and Barret, L.B. (2012). Out of Sight but Not Out of Mind: Unseen Affective Faces Influence Evaluations
 and Social Impressions. Emotion, 12(6), 1210-1221.
Dennis, A.R., Clay, P.F. and Ko, D.K. (2017). From Individual Cognition to Social Ecosystem: A Structuration Model of Enterprise Systems Use. AIS Transactions on Human Computer Interaction, 9(4), 301-338.
Postmes, T., Haslam, S. A., & Jans, L. (2013). A single‐item measure of social identification: Reliability, validity, and utility. British Journal of Social Psychology, 52(4), 597-617.
Venkatesh, V., Morris, M.G., Davis, G.B. and Davis, F.D. (2003). User Acceptance of Information Technology: Toward a Unified View. MIS Quarterly, 27(3), 425-478
